# Development of a web-based assessment tool that evaluates the meal situation when a child has a percutaneous endoscopic gastrostomy

**DOI:** 10.1186/s12887-019-1447-1

**Published:** 2019-03-11

**Authors:** Margaretha Jenholt Nolbris, Ann-Louise Gustafsson, Carina Fondin, Karin Mellgren, Stefan Nilsson

**Affiliations:** 1 Institute of Health and Care Sciences, Sahlgrenska Academy, University of Gothenburg, Box 457, 405 30 Gothenburg, Sweden; 2 Department of Paediatric Cancer Centre, The Queen Silvia Children’s Hospital, Gothenburg, Sweden; 3 Department of Paediatrics, Institution for Clinical Sciences, Sahlgrenska Academy, University of Gothenburg, Gothenburg, Sweden

**Keywords:** Cancer, Child, Gastrostomy tube, Web tool

## Abstract

**Background:**

Children with cancer often suffer side effects from their treatment, for example nausea and vomiting, which can lead to malnutrition. If a child cannot eat orally, a percutaneous endoscopic gastrostomy (PEG) can improve his or her well-being, psychosocial development and growth by enabling the supply of nourishment and facilitating the administration of necessary medicines. Few data exist on children’s comfort when using a PEG. The aim of this study was firstly to develop three versions of a web-based assessment tool in which children, families, and healthcare professionals would be able to register their observations and assessments for evaluating the meal situation when a child has a PEG and secondly to validate the content of the tool.

**Methods:**

A qualitative design was chosen with purposive sampling of participants. Five children with cancer, five parents, five registered nurses and five paediatricians participated first in an interview and then in a member check of the web-based tool. The data were analysed with manifest qualitative content analysis.

**Results:**

The results highlighted four categories of issues which needed to be revised in the web-based tool: words which were difficult for the participants to understand, items which contained several questions, items which needed to be split into more items to be answerable and the layout of the questionnaire. The web-based tool was revised according to the categories, and then a member check evaluated and finally confirmed the revisions.

**Conclusions:**

A web-based tool may be able to evaluate the meal situation when a child with cancer has a PEG. The tool may be able to detect early failures of the PEG, facilitating early action from the healthcare professionals in supporting the child and his or her parents in their care of the PEG. In the long run, this web-based tool may also be able to increase the quality of care of children living with a PEG.

**Electronic supplementary material:**

The online version of this article (10.1186/s12887-019-1447-1) contains supplementary material, which is available to authorized users.

## Background

If a child cannot eat orally for some reason, a gastrostomy is sometimes necessary to optimize the child’s well-being, growth and development. The purpose of the child’s percutaneous endoscopic gastrostomy (PEG) is to ensure that his or her need for nourishment is met [1]. A PEG can be an option to facilitate the supply of food and medicines, and it has been used successfully in both adult and child care [2]. The insertion of a PEG is a safe method with a low rate of serious complications [3]. PEG feeding through a gastrostomy tube has also been identified as a safe way to prevent malnutrition in children with cancer [2], but there is little data about whether it is comfortable for the child and his or her parent to use a PEG, and the frequency of complications is seldom reported.

In research, parents of children with a PEG have reported an easier everyday life because they felt that the child was less stressed. They also felt a sense of freedom after the child had had the PEG fitted [4–6]. When the parents knew that the child was receiving sufficient food and medicines with the PEG, they also reported a sense of security [7]. However, the parents’ responsibility to take care of the child’s PEG also had negative social and emotional consequences [8], and they reported concern that the PEG may give their children a poor health-related quality of life [9].

A PEG can be used to facilitate the care of children who are treated for cancer. A child with cancer receives treatment that is very intensive, often consisting of a combination of chemotherapy, radiation and surgery [10, 11]. Common side effects of the treatment are nausea, vomiting and altered taste sensations. This often has a negative impact on food intake, resulting in malnutrition for many children with cancer [10–12]. A nutrient intake which is too low during the child’s cancer treatment can influence the results negatively [10]. Accordingly, when a child is diagnosed with cancer, it is important to maintain a good nutritional status to pursue optimal treatment and maximize the child’s chances of survival. Malnutrition in children with cancer can be prevented with the help of a PEG [10, 13].

Only a few studies have explored the parents’ and the children’s experiences of using a PEG in paediatric oncology treatment. Nor have the few published studies on the effects of using a PEG indicated an increased risk of medical complications from its use [11]. More research is therefore needed to explore children’s, parents’ and healthcare professionals’ experiences of using a PEG as well as their reports of observed complications. A web-based tool may be helpful in fulfilling these requirements.

No web-based tools have hitherto been validated for evaluating the care of a gastronomy port. Such a tool could support the child, parents and healthcare professionals in the care of the PEG.

The symptom management model can be used to explore the child’s experiences of symptoms. This model identifies three domains: person, health/illness and environment. The model highlights the fact that assessments of symptoms should be based on self-reports, as individual preferences have an impact on the way symptoms are experienced. Different symptoms can also be expected, depending on the child’s diagnosis, for example when the child in paediatric cancer care is fitted with a PEG. The child’s symptoms are also influenced by the environment; for example a meal situation at the hospital is probably different from a meal situation at home or in school. The model can support the child, family members and healthcare professionals involved in paediatric cancer care in assessing and managing symptoms which appear when the child has a PEG [14].

The aim of this study was firstly to develop three versions of a web-based assessment tool in which children, families, and healthcare professionals would be able to register their observations and assessments for evaluating the meal situation when a child has a PEG and secondly to validate the content of the tool.

## Method

This study used qualitative design and was conducted in three steps from January 2013 to August 2017. The step one, two, and three were conducted sequentially.

In the first step, data were collected using individual interviews with the group of experts (see beneath) to find important items for a web-based tool. The goal for this tool was to evaluate the meal situation based on the symptom management model, i.e. things concerned with complications and the functional effectiveness of the PEG.

The participants in the second and third steps were children, parents, registered nurses, and paediatricians who were twice interviewed individually, using a ‘think-aloud’ method, to establish the content validity of the web-based tool [15].

In the second step, the purposive sampling of participants was chosen. The method of using the ‘think-aloud’ interviews in the validation process was useful in capturing the participants’ views of the tool [16]. The results of the interviews were analysed with a manifest content analysis [17].

**Fig. 1:**
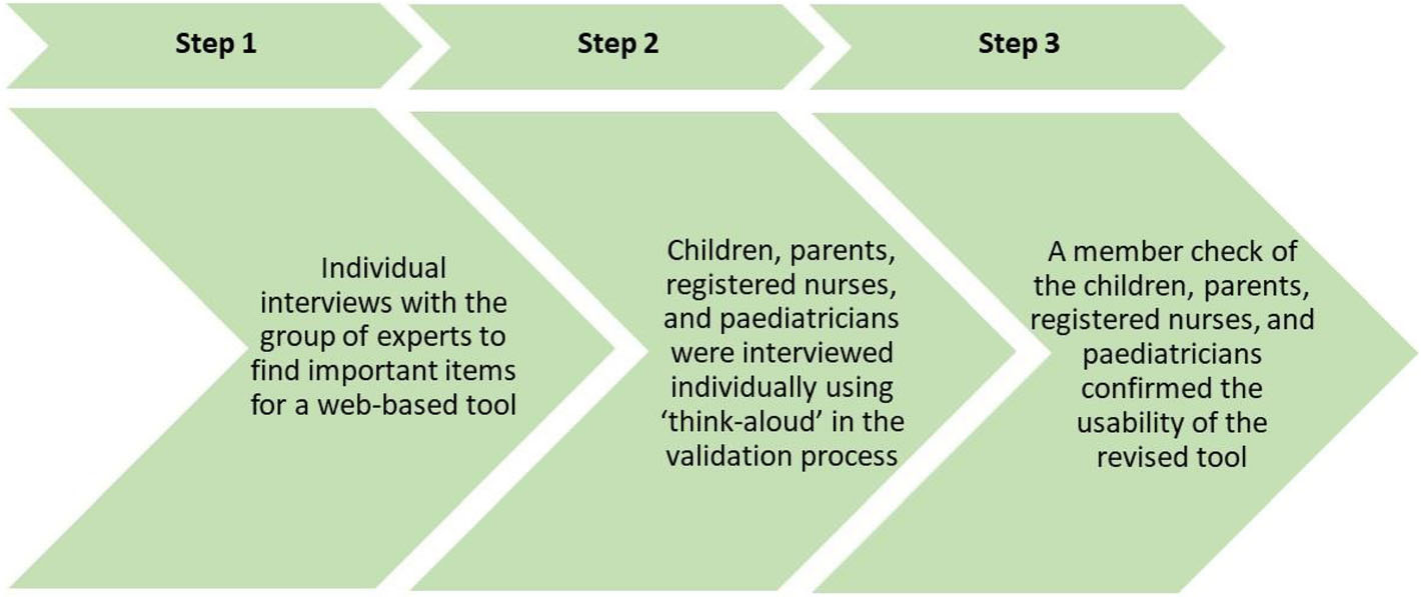
Step one, two, and three in the development of the web-based tool

In the third step, the revised form of the web-based tool was confirmed by a member check, sharing the entire revised tool with the participants [18]. The purpose was to explore whether the results were in agreement with the participants’ thoughts [19] Fig. 1.

### Setting

The expert group consisted of seven experts in the fields of paediatric oncology, surgery, nutrition and IT. This interdisciplinary group covered four professions. Four paediatric nurses skilled in oncology (CF, MJN, ALG, SN) participated, two of whom had a PhD (MJN, SN). A paediatrician and professor (KM) who specialized in oncology participated for the purpose of adding important items about the medical support. A physician and PhD, Gunnar Göthberg, who specialized in paediatric surgery, participated for the purpose of adding important items about the PEG. Finally, technical assistance was provided by an IT developer/designer, Richard Ingemannsen, and an illustrator, Gunilla Wärnström (GW), for the drawings of the PEG.

### Development of the tool

The aim of this project was to develop three versions of a web-based assessment tool in which children, families and healthcare professionals would be able to register their observations and assessments of the PEG.

Step one of this project was to collect and interpret the experience of the healthcare professionals in the research team, using individual interviews. The purpose of this step included identifying and formulating items which would identify complications and effects caused by using a PEG. The project group discussed and tested ideas for various items which could identify and report issues with the PEG. As the items were tested, the experts continued to develop images which could support the assessment of the skin around the PEG as well as follow-up questions, depending on the status of the skin. The development of images started from photographs of patients, which clearly showed skin problems with the PEG. A professional illustrator (GW) created four phases of images of the PEG. Both the items and the images were then available for the expert group.

### The tool

Following discussions, an expert group developed a web-based tool according to the symptom management model, i.e. with five items of biographical information, two items evaluating the use of the PEG, three items evaluating pain, ten items evaluating the status and comfort of the PEG, seven items evaluating nutrition, one item on how the child was feeling that day and one item for other comments.

There were a total of 29 items, and the tool took approximately 10–15 min to fill in. We suggest that this 29-item web-based tool be presented at the item level to best determine the child’s experience of the meal situation. Some of the items consisted of a dichotomized scale (i.e. yes or no), while others used a Likert or numeric rating scale. The child’s well-being was rated on a six-graded faces (smileys) scale. The purpose was to include all the factors which might influence a meal situation for a child with a PEG. Items that include the Likert scale (No; Yes, sometimes; Yes, often; Yes, always) can be calculated as 0–3, the numeric rating scale can be calculated as 0–10, and the faces (smileys) scale can be calculated as 1–6.

### Validation of the tool

The think-aloud method was used to validate each item in the web-based tool [15]. The purpose of the method was to evaluate the content validity. The participants were informed of the purpose of the think-aloud method, and each participant individually included their thoughts about and interpretation of each item in the web-based tool, while a researcher wrote down what the participant said [16].

The think-aloud method has previously been used in different types of research, for example in conjunction with emergency department triage [20], mental health nursing [21] and critically ill patients, to study cognitive error [22].

### Participants

The participants who evaluated the content validity of the web-based tool were recruited by one of the nurse coordinators at the Paediatric Cancer Centre at the Queen Silvia Children’s Hospital in Gothenburg, Sweden. The participants consisted of five children with cancer, five parents of children with cancer who had a PEG (not the participating children) and ten healthcare professionals who worked at the Paediatric Cancer Centre (five registered nurses and five paediatricians). The healthcare professionals each had a minimum of ten years of experience in paediatric cancer care (Table 1).

**Table 1:** Description of the participants

Participants		Male	Female	Has had a PEG	Age
	N	N	N	N	Range
Child with cancer	5	2	3	2	9.6–17.2 years
Parent of a child with cancer who had a PEG	5	1	4		
Registered Nurse (RN)	5	0	5		
Paediatrician	5	2	3		

### Data collection

The qualitative interviews were collected from April 2016 to March 2017. All participants in the second and third steps of the development took part twice in the study, first in an individual interview and then in a member check of the web-based tool (Table 2).

**Table 2:** Items which were revised after the member check

Participants	Number of comments (N/Total)	Descriptions of comments
Child with cancer	0/5	–
Parent of a child with cancer who had a PEG	0/5	–
Registered Nurse (RN)	1/5	Item 7, write “Soaking up the slime”
Paediatrician	1/5	Item 6, add the choice “I don’t use the percutaneous endoscopic gastrostomy”

The interviews were first conducted individually by two of the researchers (CF, MJN). The parents and the healthcare professionals were interviewed by telephone by one of the researchers (MJN), and the children were interviewed face-to-face by the other (CF). The interviews lasted between 10 and 15 min (Md = 12.5 min).

Second, all participants were invited to a member check. A paper version of the revised web-based tool was presented. The tool was sent to each participant with a letter in which they could agree or not agree to participate. If they agreed, they sent back their review of the web-based tool. The participants thought aloud about how and why they answered the items in a particular way. They told a researcher what they thought and felt about the issues. This way, the web-based tool could be evaluated as to whether the items were worded correctly, the response options were adequately thought through and arranged, and the items generated the answers which were intended [23].

### Analysis

The data from the interviews in the second step were analysed with a manifest qualitative content analysis. A manifest analysis describes the visible and obvious components in the content of the data [17, 24] to draw a systematic conclusion from the answers to each item for the purpose of responding to the research question [25]. In the first step, two of the authors (MJN, SN) individually read the text to gain an impression of it as a whole. In the second step, the authors individually coded the directly expressed thoughts and interpretations of the participants. In the third step, the codes were sorted into categories. In the fourth and last step, the authors discussed the findings until agreement was reached [17].

The data from the third step in the data collection, the member check, were not analysed using a content analysis. The participants could instead revise the items directly during the member check, if they thought that the authors had misunderstood their thoughts and interpretations.

## Results

### The interviews in step 2

The interviews in the second step resulted in four categories, which highlighted text which needed to be revised in the web-based tools.

### Words which were difficult for the participants to understand

The word ‘rub’ was difficult for young children to understand (i.e. *Do children understand what ‘rub’ means and can the word be explained or changed;* Parent 4). Another word which was difficult to understand was ‘entrance’ (*Does everyone know what is meant by the ‘entrance’ and could this be clarified;* Parent 1). The item about infection contained the word ‘infection’, and one participant replied, *Does everyone know what an ‘infection’ is* (RN 4).

The item about Lactobacillus plantarum used the trademark of a product, i.e. ProViva®. Using a trademark can be problematic, because several products may have the same content, and one participant asked, *Does everyone know what ProViva is* (Patient 2).

Some found it tricky to interpret the meanings of words; for example the location and intensity of pain was not understood (i.e. *Should it say how much pain you have;* Patient 2). Another participant responded, *Where is the pain,* e.g. *the stomach, the area around it or directly in it* (Paediatrician 5).

The item about the status of the PEG required an explanation of the meaning of the words ‘swelling’ and ‘redness’ (*A bit convoluted what the treatment is and what it is that has increased the swelling and redness;* Paediatrician 5).

When it came to the questions about food, it was unclear whether the item meant *food or probe nutrition* (Paediatrician 3). According to the participants, it was also difficult to *estimate how much food you eat in ml* (Patient 1). The item also needed to highlight that it is the *mouth* (Patient 1). In the same way, it was important in item 11 to *mark PEG/bottom* (Patient 1).

It could be tricky to understand how *you get nutrition through central venous catheter/a venous port* (RN 4) and *it can be difficult to estimate* (Paediatrician 2).

### Items that contained several questions

Some items contained *several questions in one question* (RN 4). The item about infection needed to be split into three different items, i.e. *Do you have a fever? Do you get antibiotics? If yes, is this by mouth, the PEG or intravenously?* (Paediatrician 3). An item could also be added with *cause of the fever* (Paediatrician 4).

One item contained both ‘soft’ and ‘supple’. This means that it contained *two questions on one issue* (RN 4). Another participant suggested that *maybe there should be an option such as not up to date or similar* (Paediatrician 4).

Another item also contained two questions, i.e. ‘Do you like to eat food, and does it taste good?’ (*Two questions in one, write two questions;* Paediatrician 5).

Some of the items may have more than one answer, and the person who fills in the tool needs to be informed about this (*Write that more than one option may be chosen;* Paediatrician 1).

### Items that needed to be split into more items to be answerable

The item about whether the child could sleep without discomfort from the PEG needed revision. The participants suggested that the item could be split into two, i.e. *add a question about the reason, for example infection* (Parent 6).

One item only gave the option to reply *daily*, but it should be expanded to *daily, once a day or several times each day* (Paediatrician 5).

One item asked how much food/probe nutrition the child took orally each day. This item needed to have an item added about *drinks* (RN 5) and also *multiple choices of meals* (Paediatrician 5).

One item asked about the child’s use of the PEG. The participants suggested that the items *suck out mucous* (Parent 6), *nothing at all* (Paediatrician 5) and *one option for each answer* (Paediatrician 5) should be added.

### The layout of the questionnaire

The item about well-being used smileys, which served the purpose of symbolizing the child’s emotions. These could be hard for young children (i.e. younger than 5 years old) to understand (i.e. *Young children may find it difficult;* RN 5).

### The member check in step 3

When the member check was conducted, eighteen of the participants were satisfied with the tool, and another two suggested some clarifications of the items (Table 2).

## Discussion

It is important that the items in the tool are clear and easy to understand. The results showed an initial validity of the web-based tool, which should minimize the risk of a high number of invalid answers. This is especially important to consider when a wide range of participants should be able to reply. For example, age and maturity influence young children’s ability to answer appropriately if the language is not adapted or supported with images [19]. This initial validity study tried to evaluate whether the items covered all the aspects of a meal situation, i.e. whether the tool reached content validity [26]. The purpose was also to create a tool which would catch the symptoms related to the use of a PEG by using the domains of the symptom management model (person, health/illness, environment) [14].

This web-based tool was developed in an expert group which highlighted the complications and feasibility of using a PEG. The final version of the tool includes many of the most frequently reported complications in the literature. One of these complications when using a PEG is inflammation during periods of neutropenia [27], and oral mucositis affects the majority of the children undergoing haematopoietic stem cell transplantation [28]. The impact of these complications from PEG feeding is unknown, and research is lacking in the literature.

The item which involved a faces (smileys) scale was easy to use in the interviews. It has been shown that faces make it easier for children to understand and communicate the meaning of emotions [29].

Other items needed to be specified after the interviews, such as the location of pain, which was implemented as a specific item. In other research, children aged 5–14 years have been able to indicate reliably where it hurt after laparoscopic surgery [30]. Clarification was also needed on whether some of the items were associated with food ingested by mouth or through the probe.

The results also highlighted some words which were difficult to understand, and the meanings of these words needed to be made clearer. These words may otherwise influence the validity of the tool, as it would not be known whether the participant has understood the content. This could apply to young children, in particular, who may find it difficult to interpret certain items. Earlier research has found that children under 5 years old have difficulties reporting non-present and hypothetical situations [31].

Some items also included several questions in one, which may also influence the validity of the tool. It is important to know which question the participant has answered. When items are developed for a web-based tool, it is important to ensure that the best wording is used in the final version and that poorly worded items are revised [32].

The web-based tool developed in this study was seen as usable, and the participants found that the time it took to complete the tool was acceptable. From this point of view, the web tool may be suitable for use when a child has a PEG. However, this is the first step in the validation of the web-based tool, and more studies are needed to further develop and validate the items.

This study has several limitations, including the fact that the results only focus on a few aspects of the validity of this web-based tool. This initial validation study will need to be followed up with other studies with more data on reliability and validity, and it will be valuable to evaluate the construct validity in a larger sample of participants. Of the five children who participated, only two have had PEG. This study had few participants, but the sample size can be sufficient for this type of an initial think-aloud test [16].

## Conclusion

We developed and tested a web-based tool to evaluate the meal situation when a child with cancer has a PEG (Additional file 1). While the further validation studies are necessary, we demonstrated an initial validity of the tool, which may be able to detect early failures of the PEG. Such early knowledge of PEG failure would facilitate timely action from healthcare professionals in supporting the child and his or her parents in their care of the PEG. In the long run, this web-based tool may also be able to increase the quality of care of children living with a PEG.

## Additional file

Additional file 1: The child/adolescent version of the web-based tool CaAMeal. This is the child/adolescent version of the CaAMeal, which also has a by proxy version for parents and another for healthcare professionals. (DOCX 2111 kb)

## Abbreviations

PEG: Percutaneous endoscopic gastrostomy
RN: Registered nurse
