# Supplementary material for: Development of a web-based assessment tool that evaluates the meal situation when a child has a percutaneous endoscopic gastrostomy
Source: BMC Pediatr. 2019 Mar 11;19:76. doi: 10.1186/s12887-019-1447-1 (PMC6410499; doi:10.1186/s12887-019-1447-1)
Supplement: Supplementary file 1 — The child/adolescent version of the web-based tool CaAMeal. This is the child/adolescent version of the CaAMeal, which also has a by proxy version for parents and another for healthcare professionals. (DOCX 2111 kb) [file 12887_2019_1447_MOESM1_ESM.docx]

**Meal situation for a child or adolescent who has a percutaneous endoscopic gastrostomy (CaAMeal)**

**Code……**

*Assess your situation in the last seven days (unless a different period is specified)*

**Date (day/month/year, for example 05/10/2017)**

**……………………………..**

**I have one of the following percutaneous endoscopic gastrostomies:**

***Choose one or more options***

- Mic-Key
- Mini One
- Other………….

***Biographical information***

1. **Who is filling in the web-based tool?**

- Child/adolescent
- Child/adolescent together with his or her legal guardian
- Child/adolescent together with his or her health care professional
- Child/adolescent together with another person; please specify

**…..………………………………………**

1. **You are a**

- Boy
- Girl

1. **Your date of birth (day/month/year, for example 05/10/2017)**

**………………………………………………………..**

1. **Where are you right now?**

- In my home
- At my local hospital
- At the Paediatric Cancer Centre
- Somewhere else; please describe……………………………………….

1. **What is your current weight?**

**……………………………..**

***Use of the* *percutaneous endoscopic gastrostomy***

1. **How often do you use your percutaneous endoscopic gastrostomy?**

- More than 4 times a day
- 3-4 times a day
- 1-2 times a day
- Once a week
- I don’t use the percutaneous endoscopic gastrostomy

1. **For which activities do you use your percutaneous endoscopic gastrostomy?**

***Choose one or more options.***

- Probe nutrition
- Medicine
- Soaking up the slime
- Other; please describe ……………………………………….
- I don’t use the percutaneous endoscopic gastrostomy

***Pain***

1. **Does your percutaneous endoscopic gastrostomy hurt?**

- Yes, always
- Yes, often
- Yes, sometimes
- No
- I don’t know

**Assess the intensity of your pain today on a scale of 0-10 ……………….**

(where 0 is no pain and 10 is the worst pain imaginable)

**Please write down your own answer**

**………………………………………………………………..**

1. **Does it hurt inside in the place where you have the** **percutaneous endoscopic gastrostomy?**

- Yes, always
- Yes, often
- Yes, sometimes
- No
- I don’t know

**Assess the intensity of your pain today on a scale of 0-10 ……………….**

(where 0 is no pain and 10 is the worst pain imaginable)

**Please write down your own answer**

**………………………………………………………………..**

1. **If you are in pain today, please mark on the pictures where your pain is.**

- **If you not are in pain, please mark this circle.**


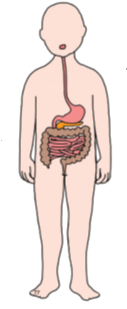

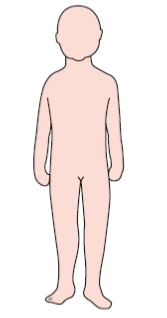


***Status and comfort of the percutaneous endoscopic gastrostomy***

1. **Does the percutaneous endoscopic gastrostomy hinder you when you are playing or doing other activities?**

- Yes, always
- Yes, often
- Yes, sometimes
- No
- I don’t know

1. **Is the percutaneous endoscopic gastrostomy soft?**

- Yes, always
- Yes, often
- Yes, sometimes
- No
- I don’t know

1. **Is the percutaneous endoscopic gastrostomy smooth?**

- Yes, always
- Yes, often
- Yes, sometimes
- No
- I don’t know

1. **Does the percutaneous endoscopic gastrostomy create pressure somewhere on your body?**

- Yes, always
- Yes, often
- Yes, sometimes
- No
- I don’t know

1. **Does the hole, i.e. the percutaneous endoscopic gastrostomy, leak?**

- Yes, always
- Yes, often
- Yes, sometimes
- No
- I don’t know

1. **Can you sleep with the percutaneous endoscopic gastrostomy?**

- Yes, always
- Yes, often
- Yes, sometimes
- No
- I don’t know

1. **What does your percutaneous endoscopic gastrostomy look like today?**

- Granuloma

- Infection

- Redness

- Not irritated

1. **Do you currently have an infection in the percutaneous endoscopic gastrostomy?**

- Yes
- No
- I don’t know

1. **If you have an infection, are you receiving any treatment?**

- If yes, please describe your treatment **……………………………………….**

**………………………………………………………………………..**

- No
- I don’t know

1. **Do you have a fever, 38 degrees Celsius or more, at the time of filling in this web-based tool?**

- Yes
- No
- I don’t know

***Nutrition***

1. **a) Do you eat food orally?**

- Yes, always
- Yes, often
- Yes, sometimes
- No
- I don’t know

**b) How much food do you eat in general?**

**Please answer in millilitres or grams**

**Please write down your own answer**

**………………………………………………………………..**

**c) Do you like eating food?**

- Yes, always
- Yes, often
- Yes, sometimes
- No
- I don’t know

**d) Do you think food tastes good?**

- Yes, always
- Yes, often
- Yes, sometimes
- No
- I don’t know

1. **a) Do you only drink liquid orally?**

- Yes, always
- Yes, often
- Yes, sometimes
- No
- I don’t know

**b) What do you drink?**

**Please write down your own answer**

**………………………………………………………………..**

1. **Do you get fruit juice daily that contains bacteria, i.e. Lactobacillus plantarum, such as ProViva^®^?**

- Yes, always
- Yes, often
- Yes, sometimes
- No
- I don’t know

1. **Do you only get food through your percutaneous endoscopic gastrostomy?**

- Yes, always
- Yes, often
- Yes, sometimes
- No
- I don’t know

1. **Do you get food both orally and your percutaneous endoscopic gastrostomy?**

- Yes, always
- Yes, often
- Yes, sometimes
- No
- I don’t know

1. **Do you get any nutrition through your central venous catheter (CVK)/central venous port?**

- Yes, always
- Yes, often
- Yes, sometimes
- No
- I don’t know

1. **a) Do you use your percutaneous endoscopic gastrostomy for things other than food and medicines?**

- Yes, always
- Yes, often
- Yes, sometimes
- No
- I don’t know

**b) If yes, how do you use your percutaneous endoscopic gastrostomy?**

**Please write down your own answer**

**………………………………………………………………..**

***Well-being***

1. **How do you feel today?**

**As well as choosing a smiley, please write down how you feel today.**

**…………………………………………………………………………………………………**

**…………………………………………………………………………………………………**

***Other things***

1. **Do you have any other comments?**

**………………………………………………………………………………………………....**

**…………………………………………………………………………………………………**

**…………………………………………………………………………………………………**
